# Addressing missed opportunities for cervical cancer screening in Nigeria: a nursing workforce approach

**DOI:** 10.3332/ecancer.2022.1373

**Published:** 2022-04-11

**Authors:** Elvis Anyaehiechukwu Okolie, David Aluga, Seun Anjorin, Felicity Nneoma Ike, Ekene Moses Ani, Blessing Ifeoma Nwadike

**Affiliations:** 1 School of Health and Life Sciences, Teesside University, Middlesbrough TS1 3BX, UK; 2 Warwick Centre for Global Health, Division of Health Sciences, University of Warwick, Coventry CV4 7AL, UK; 3 Department of Public Health, University of Calabar, Calabar 540271, Nigeria; 4 Department of Microbiology, University of Ibadan 200284, Nigeria; a https://orcid.org/0000-0003-2283-8077; b https://orcid.org/0000-0001-8133-3525

**Keywords:** Uterine cervical neoplasms, early detection of cancer, nurses, health personnel, Nigeria

## Abstract

Cervical cancer is the commonest gynaecological cancer affecting women, especially in low and middle-income countries (LMICs). Despite the availability of evidence on multiple prevention pathways, including vaccination and screening, the cervical cancer burden continues to increase, especially in LMICs. This disease typifies health inequality as more than 85% of related morbidity and mortality occur among women of low socio-economic status residing in developing countries. In Nigeria, cervical cancer is the second leading cause of cancer morbidity and mortality. Sadly, Nigeria lacks a tailored cervical cancer control policy or population-based screening programme which is recommended. Consequently, existing screening services are opportunistic, sparsely distributed and have reached less than 9% of eligible Nigerian women. This article highlights the current status of cervical cancer screening in Nigeria, contextualises the role of female nurses and proffers novel approaches to address missed opportunities for screening by leveraging the nursing workforce.

## Introduction

Cervical cancer is the commonest gynaecological cancer and represents a public health setback [1]. This is despite the abundance of evidence showing that cervical cancer is a slow-progressing disease and can be prevented by multiple interventions – vaccination, screening and treatment [2, 3]. Annually, an estimated 570,000 cases and over 311,000 deaths from cervical cancer occur [4]. Unfortunately, cervical cancer is a disease of inequality as more than 85% of related morbidity and mortality occur among women of low socio-economic status residing in low and middle-income countries (LMICs) [5, 6]. Despite the prospects offered by the human papillomavirus (HPV) vaccine, issues such as the inability of vaccines to protect against all HPV strains, high cost especially for poor countries and inequalities in vaccine access make screening a go-to in the continuum of efforts to eliminate cervical cancer, especially in LMICs [7, 8]. The level of progress in the implementation of the organised population-based cervical cancer screening (CCS) programme has been linked to the disparities in the burden of cervical cancer between high-income and the world’s poorest countries [1, 9].

Cervical cancer ranks only behind breast cancer as the foremost cause of cancer burden in Nigeria, with an estimated 14,943 cases and 10,400 deaths occurring in 2018 [4]. Additionally, it is now projected that over 53 million Nigerian girls and women above the age of 15 will be at an increased risk of developing the disease if deliberate prevention and control measures are not implemented [10]. These estimates may mirror an ‘iceberg phenomenon’ of the actual burden of cervical cancer in Nigeria due to poor data quality and reporting, lack of national prevalence studies and an inadequate number of cancer registries [11, 12]. Nevertheless, cervical cancer has an enormous socio-economic impact on Nigeria. By affecting Nigerian women in their prime age, cervical cancer contributes to poverty and impedes economic growth [13, 14].

In response to the unacceptable burden of cervical cancer, especially in LMICs, the 90–70–90 target to be met by 2030 towards the global elimination of cervical cancer was adopted [1]. This target serves as a rallying call for countries to fully vaccinate 90% of girls against HPV, screen 70% of women by 35 years and again by 45 years of age and 90% of women with precancer or invasive cervical cancer treatment [1]. Meeting these targets in Nigeria will rely on a concerted health system policy and programme that are driven by a strong political commitment [14, 15]. Therefore, this article explores a nursing workforce approach towards tackling missed opportunities for CCS and reducing the burden of the disease in Nigeria.

## Cervical cancer screening outcomes in Nigeria

Nigeria runs a three-tier health system across the governance levels (federal – tertiary healthcare; state – secondary healthcare; and local government – primary healthcare) and CCS services are expected to be provided at all levels [16]. On the contrary, CCS services in Nigeria are opportunistic, sparsely distributed and are mostly accessed in some secondary and tertiary health institutions [9, 16, 17]. Such an opportunistic approach has only reached less than 9% of eligible Nigerian women with CCS services, despite the increasing burden of the disease. [17] Despite the overall poor uptake of CCS services, previous studies further highlight wide disparities in uptake due to differences in women’s socio-economic status. For instance, Eze *et al* [18] found that screening uptake among rural women was as low as 0.6% compared to 10.2% observed by Hyacinth *et al* [19] among women in the urban area.

A plethora of reasons have been adduced for Nigeria’s poor CCS rates. Nigeria’s weak health system and lack of a cervical cancer control policy that facilitates a roadmap for the country’s fight against cervical cancer are considered principal problems [13, 14]. Similarly, factors such as poor awareness, low-risk perception, poverty, lack of female providers, fear of positive screening result and sociocultural norms contribute immensely to poor uptake of CCS services among Nigerian women [15, 20].

While several CCS methods exist – pap smear, HPV DNA test and visual inspection with acetic acid (VIA) – issues such as paucity of resources, cost of implementation and follow-up challenges remain significant problems in LMICs, including Nigeria [3, 7]. Moreover, the ‘See and Treat’ approach using VIA that mostly requires fewer resources, a single visit and allows for immediate treatment has been recommended as a panacea to Nigeria’s CCS challenge due to its cost-effectiveness [3, 21]. Regrettably, Nigeria is yet to make progress in the implementation of such recommendation for the prevention of cervical cancer [13, 14].

As Nigeria relies on opportunistic screening, emphasis must be given to effective measures that leverage every eligible woman’s contact with the health system to provide life-saving CCS services [14, 16]. Arguments for the integration of CCS services into existing health delivery structures have been made by academics and health stakeholders. Evidence suggests that significant progress in CCS screening outcomes can be achieved even in LMICs upon the successful integration of CCS and treatment into the general healthcare pathway [22, 23]. Service delivery points such as family planning clinics, antenatal visits and maternal and child health weeks have been recommended [22]. Integration not only ensures that women are offered an array of services but also stimulates improved uptake, efficient use of scarce resources, programme sustainability and wider coverage of underserved women [22, 23]. Nevertheless, service integration may become counterproductive or fail to meet expectations in the absence of clearly articulated operational plans and strategies [24]. These operational strategies may include staff training and supervision, adjusting existing service delivery plans, aligning coordination and financing mechanisms with service integration objectives and ensuring an effective supply chain system [22, 24]. Even where CCS services have been reported to be integrated in Nigeria, missed opportunities are observed as only a handful of women have been screened [25]. For an already overstretched health workforce, especially nurses, an additional mandate to provide CCS services may be burdensome. Therefore, the development of a comprehensive CCS service integration plan with a cross-cutting focus on health system components, especially human resources, may improve Nigeria’s CCS narratives.

## Spotlight on female nurses’ role in cervical cancer screening in Nigeria

The role of health workers, especially female nurses, in tackling Nigeria’s rising cervical cancer burden has been a vital part of ongoing calls to maximise opportunistic screening activities [15, 16]. Traditionally, female nurses, including midwives, play a critical role in the delivery of reproductive health services, including CCS services, to eligible women [26–28]. For high-income countries that have an organised population-based CCS programme, the contributions of nurses have been recognised as an important factor to observed programme success in reducing the cervical cancer burden [26, 27]. While female nurses have been shown to provide CCS services, including treatment procedures, for women in developed country settings [26, 28], their role in CCS service delivery in Nigeria has been underexplored. Previous cervical cancer-related studies with a focus on nurses mostly explored knowledge, attitude and screening practices without assessing their involvement in CCS service delivery [29–31]. Surprisingly, in a systematic review by Okolie *et al* [15], Nigerian female health workers, including nurses, were reported to have poor screening practices as those who have never screened ranged from 45.9% to 97%. This abysmal screening rate among health workers, especially nurses who are expected to be role models in screening uptake, portends danger in reversing the disease trend.

As calls to address missed opportunities for CCS services in Nigeria continue to gain momentum, it becomes imperative to explore and scale up the role of female nurses. Nurses through their education can provide CCS services and accurate information to the women visiting the health facility [28]. Female nurses can leverage their contact with a significant proportion of women at different points of the healthcare pathway, especially for maternal and child healthcare to offer CCS services [26, 29]. Again, by virtue of their sex, female nurses are also at risk of developing cervical cancer, regardless of their profession, and must also adhere to the established prevention measures [15, 28].

Furthermore, screening recommendation which is effective in facilitating CCS service uptake is an influential role that female nurses can play [32, 33]. Musa *et al* [33] observed an almost twofold increase in CCS uptake among women who reported receiving recommendations from health providers. Similarly, 53% of women who were screened in a Lagos hospital mentioned screening recommendations from their doctors and nurses as a key driver in their decision to screen [32]. Importantly, the expected roles of female nurses in providing CCS services, including recommendation, depend on their competence and an enabling health system environment.

## Nursing-based strategies to tackle missed cervical cancer screening opportunities

Strengthening the health workforce capacity for CCS service delivery, including disease management, is an essential task towards meeting the 90–70–90 target for global elimination of cervical cancer [1]. Similarly, one of the objectives of Nigeria’s National Cancer Control Plan (2018–2022) is to ensure that 40% of health facilities are strengthened by 2022 to provide cancer screening leading to early detection [34]. However, despite having a population of over 200 million people, Nigeria has less than 1,000 gynaecologists and 100 oncologists [35, 36]. Such a low number of gynaecologists and oncologists mirrors similar staff shortages in Nigeria’s health system and represents a bottleneck in the efforts to curb cervical cancer. The shortage of female screening providers, especially nurses, in providing such an intimate screening activity that has religious and cultural implications is also a known barrier for Nigerian women [21]. Hence, approaches to improve and sustain the role of female nurses may prove effective in tackling missed CCS opportunities in Nigeria. The strategic position occupied by nurses in Nigeria’s health system, the urgent need to increase CCS rates and the success of nurse-led interventions are logical reasons to target female nurses [37]. Additionally, due to their sex and shared susceptibility to cervical cancer, female nurses may relate better with women’s experiences and be better suited to meeting their needs [26, 28]. To meet the global cervical cancer elimination targets by Nigeria, the availability and equitable distribution of competent female nurses that can provide CCS services is a viable approach [9, 37].

We propose a tripartite nursing-based approach that focuses on skills gap assessment, the development of a comprehensive human resource plan for the strategic engagement of registered female nurses in clinical and general practice as screening providers and competency-based training on CCS service delivery. First, there is limited information on the overall competence of nurses to provide cervical cancer screening in Nigeria as previous studies were limited to either a state or health facility [30, 38]. The provision of a nationally representative CCS skills gap assessment to highlight female nurses’ strengths and needs will serve as a foundation for any successful national effort to scale up opportunistic screening.

Next, a comprehensive human resource plan developed with substantial inputs from female nurses is crucial to implementing and sustaining nurse-led efforts to improve CCS uptake [24]. This plan must articulate a roadmap for engaging female nurses who are seemingly overstretched, ensure mechanisms for continuous quality improvement and steps for evaluation of nursing services. Moreover, the introduction of sustainable performance-based incentives for female nurses as part of the human resource plan will encourage them to provide life-saving CCS services, especially for underserved women who visit the health facility [39].

Finally, we recommend the implementation of dedicated competency-based training (CBT) for female nurses at all levels of care in Nigeria on CCS using the VIA method which allows for ‘See and Treat’. This training will focus on providing nurses with core skills for identifying eligible women for screening, client counselling (pre and post), provision of CCS using VIA and clinical decision-making, including referral and follow-up [39, 40]. Evidence shows that between 5 and 10 days is adequate for rapid training of health workers, such as nurses, to perform VIA and provide immediate treatment [40]. The effectiveness of the cervical cancer prevention programme in Zambia [23] and the task-shifting approach in South Africa [37] where trained nurses were central to the provision of CCS services demonstrates the feasibility of similar approaches in Nigeria. CBT will allow for a detailed acquisition of CCS knowledge, correct performance of CCS, identification and treatment of patients with positive lesions and intense evaluation to ensure minimal events of false positive or negative diagnosis [39]. Following CBT, creating a feedback mechanism on outcomes of CCS provided by nurses and routine supportive supervisory visits to health facilities to monitor nurse-led CCS services are crucial for quality assurance.

As medical knowledge continues to advance, health workers are routinely trained via a mandatory continuing professional development (MCPD) programme to ensure continuous learning, maintenance of skills and enhancement of core job competencies [41, 42]. In Nigeria, as part of the qualifications for re-licensure, nurses must attend at least one MCPD programme every 3 years [41]. Sadly, MCPD programme for nurses in Nigeria may not be prioritising cervical cancer prevention as part of its training agenda. Okolie *et al* [15] observed that very few female health workers reported MCPD as their source of cervical cancer-related information compared to the large proportion that got theirs from the media. Hence, MCPD programme could be leveraged for refresher training of female nurses as a sustainable strategy beyond CBT towards improving their competencies in providing CCS and addressing missed opportunities. Ultimately, trained female nurses will lead the integration and provision of CCS services at all levels of healthcare in Nigeria.

## Conclusion

Addressing Nigeria’s cervical cancer burden requires transformative and cost-effective strategies that will scale up CCS services, especially for the most vulnerable women. We propose a tripartite approach to leverage female nurses in tackling missed opportunities for the provision of CCS services and improving screening uptake in Nigeria. The implementation of these strategies would rely on cross-cutting factors including but not limited to strong political will, availability of resources, effective institutional leadership and support, level of cervical cancer prioritisation and a competent and motivated female nursing workforce.

## Conflicts of interest

The authors declare that they have no competing interests.

## Authors’ contributions

EAO conceptualised the study and prepared the initial manuscript draft. EAO, FNI and EMA conducted the literature search for relevant articles. DA, FNI, EMA, SA and BIN critically reviewed and edited the draft manuscript before submission. All authors reviewed and accepted the final version of the article.

## Funding

None to be declared.

## Ethical approval

Not applicable.
